# P-2183. In Vitro 425 nm Light Biomodulation Reduces Influenza and RSV Titers

**DOI:** 10.1093/ofid/ofaf695.2346

**Published:** 2026-01-11

**Authors:** Jacob Kocher, Nicole Jandick, Derry Spragion, Leslee Arwood, Rachel Roberts, Max Stockslager, P Joseph DeSena, Riley Hook, T Matthew Womble, Nate Stasko

**Affiliations:** KNOW Bio LLC, Morrisville, North Carolina; EmitBIo, Morrisville, North Carolina; EmitBio, Morrisville, North Carolina; KNOW Bio LLC, Morrisville, North Carolina; KNOW Bio LLC, Morrisville, North Carolina; EmitBio, Morrisville, North Carolina; EmitBio, Morrisville, North Carolina; EmitBio, Morrisville, North Carolina; KNOW Bio LLC, Morrisville, North Carolina; KNOW Bio LLC, Morrisville, North Carolina

## Abstract

**Background:**

The RD-X19 is an investigational medical device that targets 420 – 430 nm light to the oropharynx and surrounding tissues to treat upper respiratory viral infections. The development of the RD-X19 was translated from the biological light unit (BLU), a proprietary high-throughput *in vitro* test platform. Clinical trials have indicated that the RD-X19 device can reduce the time to sustained symptom resolution and the time to negative antigen tests in clinical subjects 40 years of age and over with mild COVID-19. Visible light impacts SARS-CoV-2 through modulation of host and viral factors; therefore, we expect 420 – 430 nm light biomodulation to be effective against other respiratory viruses, including influenza and respiratory syncytial virus (RSV).

**Methods:**

In this study, we utilized both the BLU and RD-X19 to test whether 420 – 430 nm light a) inactivated influenza A H1N1 and H3N2, influenza B, and RSV in cell-free suspensions and b) reduced viral titers in well-differentiated models of human airway epithelia (HAE). Cell-free suspensions of each virus were illuminated with 420 – 430 nm light and viral titers were determined. HAE were inoculated with virus prior to twice-daily treatment with 420 – 430 nm light. Apical washes were collected and viral titers were determined.

**Results:**

H1N1 and H3N2 and influenza type B titers in cell-free suspensions. However, 420 – 430 nm light reduced RSV titers in a dose-dependent manner. In HAE, 420 – 430 nm light significantly reduced influenza A H1N1 and H3N2, influenza B, and RSV titers supporting that host factors play role. The illumination of RSV with the RD-X19 device induced similar dependent inactivation of RSV as those observed with the BLU.

**Conclusion:**

These data demonstrate the potential of the RD-X19 as a novel treatment for upper respiratory infections regardless of the infecting pathogen. Based on the comparable benchtop findings in HAE for influenza and RSV as those in the clinically-evaluated SARS-CoV-2, we anticipate that the RD-X19 treatment should achieve clinical success against influenza and RSV and warrants further clinical investigation.

**Disclosures:**

Jacob Kocher, PhD, EmitBio, Inc.: US Patent 11,986,666/US Patent 11,980,774|EmitBio, Inc.: Stocks/Bonds (Private Company) Nicole Jandick, PhD, EmitBio: Stocks/Bonds (Private Company) Derry Spragion, M.S., EmitBio, Inc.: Stocks/Bonds (Private Company) Leslee Arwood, B.S., EmitBio, Inc: Stocks/Bonds (Private Company) Rachel Roberts, B.S., EmitBio, Inc: Stocks/Bonds (Private Company) Max Stockslager, Ph.D., EmitBio, Inc.: Stocks/Bonds (Private Company) P. Joseph DeSena, M.S., EmitBio, Inc.: Stocks/Bonds (Private Company) Riley Hook, B.S., EmitBio, Inc.: Stocks/Bonds (Private Company) T. Matthew Womble, M.S., EmitBio, Inc.: Stocks/Bonds (Private Company) Nate Stasko, PhD, EmitBio, Inc.: Stocks/Bonds (Private Company)

